# Clinically actionable cancer somatic variants (CACSV): a tumor interpreted dataset for analytical workflows

**DOI:** 10.1186/s12920-022-01235-7

**Published:** 2022-04-25

**Authors:** Turki M. Sobahy, Ghassan Tashkandi, Donya Bahussain, Raneem Al-Harbi

**Affiliations:** 1 King Faisal Specialist Hospital & Research Center-Jeddah (KFSHRC-J), Research Center, Jeddah, 21499 Kingdom of Saudi Arabia; 2 Genetic Medicine Department, College of Medicine, King Abdulaziz University (KAU), Jeddah, 7393 Kingdom of Saudi Arabia

**Keywords:** Somatic genetic variants, AMP–ASCO–CAP recommendations, Tumor site(s), Genetic variant class, Gene-tumor consensus

## Abstract

**Background:**

The recent development and enormous application of parallel sequencing technology in oncology has produced immense amounts of cell-specific genetic information. However, publicly available cell-specific genetic variants are not explained by well-established guidelines. Additionally, cell-specific variants interpretation and classification has remained a challenging task and lacks standardization. The Association for Molecular Pathology (AMP), the American Society of Clinical Oncology (ASCO), and the College of American Pathologists (CAP) published the first consensus guidelines for cell-specific variants cataloging and clinical annotations.

**Methods:**

AMP–ASCO–CAP recommended sources and information were downloaded and used as follows: relative knowledge in oncology clinical practice guidelines; approved, investigative or preclinical drugs; supporting literature and each gene-tumor site correlation. All information was homogenized into a single knowledgebase. Finally, we incorporated the consensus recommendations into a new computational method.

**Results:**

A subset of cancer genetic variants was manually curated to benchmark our method and well-known computational algorithms. We applied the new method on freely available tumor-specific databases to produce a clinically actionable cancer somatic variants (CACSV) dataset in an easy-to-integrate format for most clinical analytical workflows. The research also showed the current challenges and limitations of using different classification systems or computational methods.

**Conclusion:**

CACSV is a step toward cell-specific genetic variants standardized interpretation as it is readily adaptable by most clinical laboratory pipelines for somatic variants clinical annotations. CACSV is freely accessible at (https://github.com/tsobahytm/CACSV/tree/main/dataset).

**Supplementary Information:**

The online version contains supplementary material available at 10.1186/s12920-022-01235-7.

## Introduction

Next-generation sequencing (NGS) is a major technological advancement in biological sciences. NGS is a high-throughput, efficient and cost-effective method in contrast to single sequence or gene-by-gene techniques and it has replaced most hybridization assays for genetic variants screening and detection. NGS technology has created a multi-dimensional data space. Indeed, sequencing has allowed for the identification of new genetic determinants for multiple physiological phenotypes [1, 2]. It has quickly become a component of diagnostic services in healthcare [3]. Genetic-based disease diagnosis, prognosis and management can improve clinical outcomes and patient care [4, 5].

Genetic variants represent differences in the deoxyribonucleic acid (DNA) molecule (ISBN: 978-0-12-404748-8). A genetic variant is an observable change from the most commonly known nucleotide(s) at a given loci and could be perceived as constitutional or somatic [6]. Constitutional or germline variants occur within germ cells and may pass to offspring [7, 8]. Germline variations are used as predictive biomarkers in tumor diagnostics, for predisposition and for disease risk estimations [9–12]. Conversely, somatic variants occur post-fertilization and are cell specific [7, 8]. Cell-specific variants harbor many genomics locations including cancer driver genes [13, 14]. Cancer drivers’ genes behavior can be: tumor intensifying (oncogenes), tumor suppressing, and some genes with a dual nature. For instance, the NOTCH gene is an established tumor suppressor in many solid tumors such as hepatocellular carcinoma and non-melanoma skin cancer while it behaves like an oncogene in T-cell acute lymphoblastic leukemia [15]. This highlights the importance of the gene-tumor site dimension in identifying actionable somatic variants.

Small nucleic acid variations involving single, double or triple nucleic acid bases are more readily detectable by most NGS platforms and bioinformatics workflows compared to structural changes such as copy number variations (CNVs) or other chromosomal abnormalities. For this reason, developing “trouble-free” accumulative large somatic databases such as The Cancer Genome Atlas (TCGA) (https://portal.gdc.cancer.gov/) is feasible. The spatial catalogue of cell and tissue types in cancer genomic research has revealed the complexity of carcinogenesis and tumor heterogeneity [16].

Cancer heterogeneity is defined as the presence of a subpopulation of cancer cells with various phenotypes and genotypes that may lead to contrastive biological behaviors within the primary tumor known as intra-tumor heterogeneity. When this occurs between tumors of the same histopathological subtype, it is defined as inter-tumor heterogeneity [1, 16]. The characterization of intra-tumor heterogeneity for multiple tumor samples obtained from the same patient can be referred to as spatial heterogeneity if different cancer cells exist in the same tumor site. If different cancer cells are distantly recurrent or subsequently local in the same patient, this is referred to as temporal heterogeneity [17].

Challenges in analyzing information in cancer genomics have been addressed by the development of specific tumor databases and computational tools [13, 18–20]. A wealth of genomic data has been generated and consolidated into public repositories and has stimulated ideas from data and machine-learning researchers. The list of database examples includes the Catalogue of Somatic Mutations in Cancer (COSMIC) (https://cancer.sanger.ac.uk/cosmic) [18] and cBioPortal (https://www.cbioportal.org/) [19]. However, somatic-specific hubs may include impoverished or non-specific tumor diagnoses and lack genetic clinical annotations [21]. Subsequently, thoroughly reviewed cancer resources have been developed to provide more clinically actionable information. For instance, My Cancer Genome (https://www.mycancergenome.org) [22] and Personalized Cancer Therapy (PCT) (https://pct.mdanderson.org) are highly curated with potential clinical utility. Though, it is not readily to incorporate into analysis pipelines or available for bulk downloads.

Several in silico algorithms have been developed to measure the impact of small genetic variants on gene function [20, 23–26]. Some methods perform differently in estimating the effect of germline and somatic variants such as FATHMM-MKL [20] which could be related to a lack of cell-specific molecular background knowledge. Other predictive models do equally well with general and cell-specific variants (CADD, DANN, & ClinPred) [20, 23]. In addition, oncogenic-specific computational methods like CScape have been created to precisely evaluate the consequences of somatic variants [20]. Other methods predict the causality of genes in cancers using different molecular and genetics background knowledge. Truly, there is no gold standard for computational tools used for classifying and interpreting cell-specific variants in tumors [16, 21].

The lack of standardization in the interpretation of cancer genetic variants in clinical settings is fairly noticeable [15]; a survey of over 44 labs revealed a discernible degree of variation in the reporting and interpretation of cancer variants [21]. The tier-systems used for variants clinical interpretation were found to have unique proportions among the labs surveyed. While 40% applied tier five, 30% used tier three, and 30% implemented other classification systems. Uniformity in clinical interpretation and reporting of results among different laboratories is crucial for reaching a common standard. In 2017, a multidisciplinary working group tasked with assessing the current status of NGS–based cancer testing and establishing a standardized consensus for classification, annotation, interpretation, and reporting conventions for somatic sequence variants was convened by the Association for Molecular Pathology with representation from the American College of Medical Genetics and Genomics, the American Society of Clinical Oncology, and the College of American Pathologists [21]. Jointly the first recommendations for somatic variants interpretation in cancer was published (known as the AMP–ASCO–CAP recommendations).

**Fig. 1:**
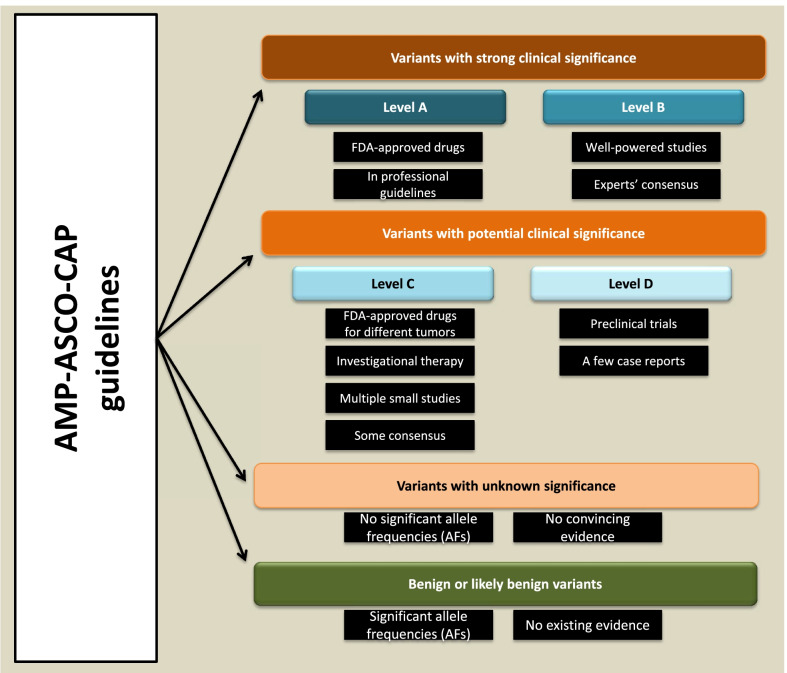
The current (2017) consensus guidelines for genetic variant interpretation in tumors

The recommendations provide a four-tier classification system for cancer genetic (somatic) variants based on the availability and significance of clinical and genomic information (Fig. 1). Class I includes variants with strong clinical significance, class II covers variants with potential clinical significance, class III is for variants with unknown clinical significance, and class IV includes variants with significant allele frequencies in the general population. The level of evidence is also catalogued into four groups. Group A contains genetic knowledge in oncological professional guidelines or therapeutic information for an FDA-approved drug for a particular type of cancer. Group B includes reported knowledge in well-powered studies with consensus (gene-tissue vector) by specialists. Group C includes knowledge about a drug investigation for a specific tumor type, an FDA-approved drug for any given tumor type, or knowledge from a few small studies with limited gene-tissue consensus. Finally, group D includes data about preclinical trials or preliminary publications with no consensus [21].

**Fig. 2:**
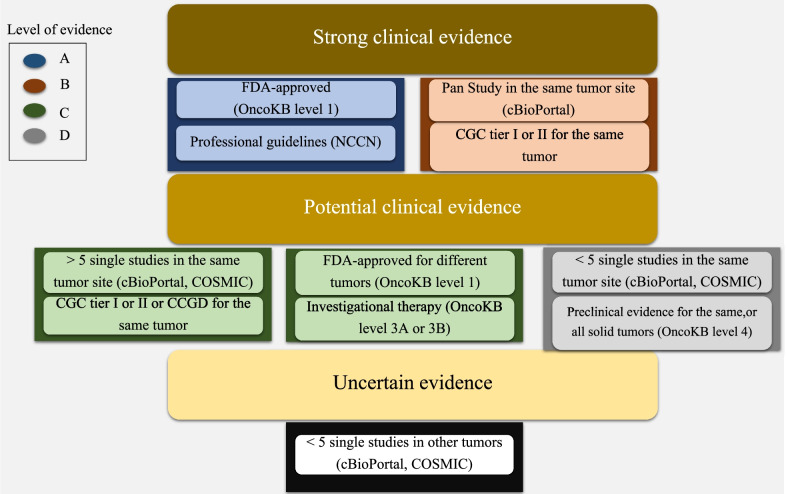
The adaptive algorithm for the AMP–ASCO–CAP recommendations

We incorporated the AMP–ASCO–CAP recommendations into a new computational method (Fig. 2). A list of genetic variants was manually curated for the clinical annotations for the method evaluation. The new classifier was applied to annotate publicly available somatic variants that are trouble-free on most NGS platforms and we developed a clinically actionable cancer somatic variants (CACSV) dataset in easily incorporated formats (JSON).

## Methods

### Level of evidence

**Fig. 3:**
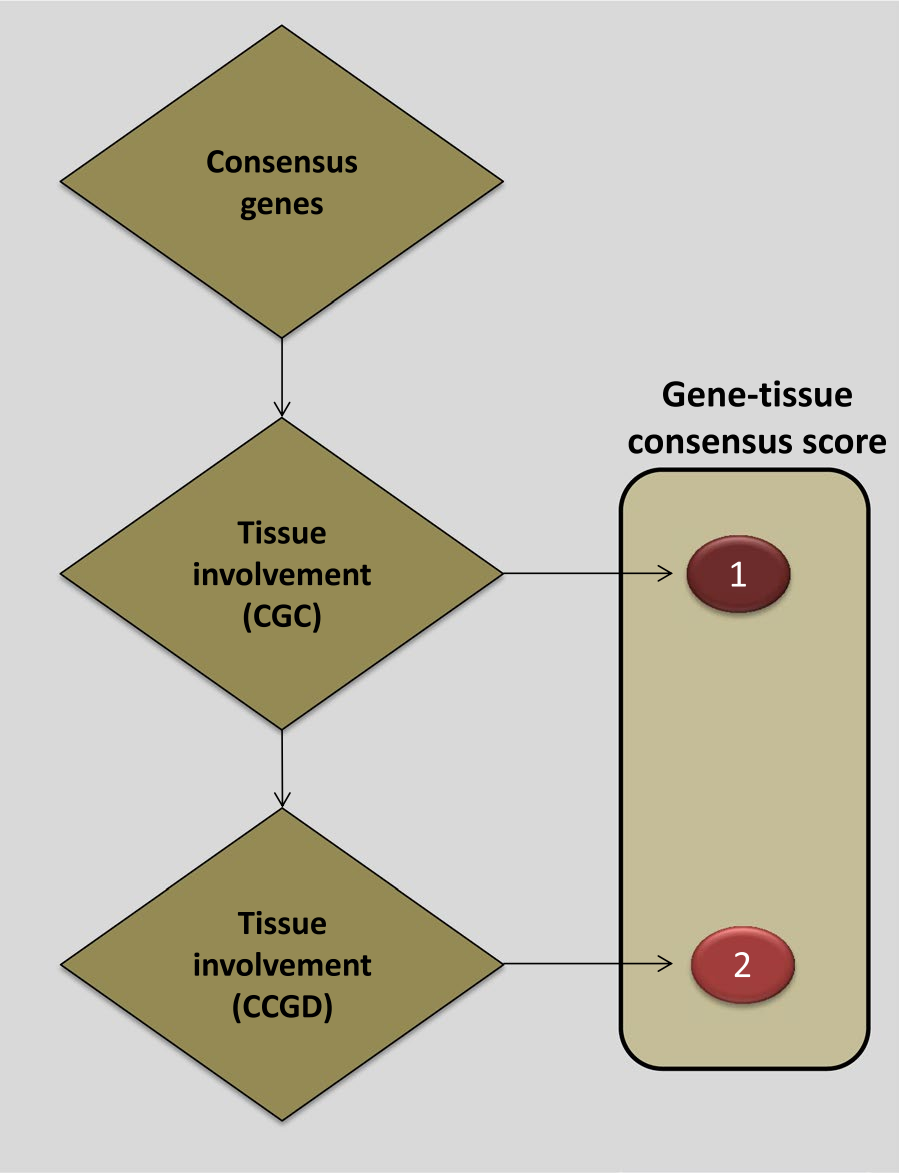
The developed algorithm for measuring cancer gene consensus in different cells of origins. CGC tiers I and II are equally ranked

Identifying the source for each evidence level is important for genetic variants classification systems. We used the National Comprehensive Cancer Network Clinical Practice Guidelines in Oncology (NCCN Guidelines®) as the professional guidelines for variants clinical information. Precision Oncology Knowledge Base (OncoKB) was our source of druggable genetic variants with approved, investigative treatments or preclinical evidence. The type and the level of supportive literature were collected from cBioPortal and COSMIC. Finally, the level of concurrence (of the gene-tissue dimension) was measured by cataloging the knowledge of genes in the Cancer Gene Census (CGC) (https://cancer.sanger.ac.uk/census) and the Candidate Cancer Gene Database (CCGD) (http://ccgd-starrlab.oit.umn.edu/) in comparison to their tissue involvement (Fig. 3, Additional file 1: Table S1).

### Text mining

Information about actionable genetic variants in the NCCN guidelines was collected manually, solely for single genetic variants (SNVs). There were non-specific descriptions for some variants in the NCCN guidelines; for instance, the NCCN panel suggested the use of KRAS activation variants (codon 12 and others) as therapeutic and prognostic biomarkers for non-small-cell lung carcinoma (NSCLC) patients. Consequently, only kinase domain KRAS variants with high confidence predictive scores (CScape) and predicted tumor drivers (intOgen) were selected. Other examples are hyper-mutated genes like the BRCAs (1/2) variants in ovarian cancers. The NCCN panel discussed the use of BRCA changes (germline or somatic) to inform maintenance therapy and TP53 variants in acute myeloid leukemia (AML) patients. The AML panel suggested the use of such variants as prognostic biomarkers. Genes known to harbor mixed genetic variants (germline and somatic) require careful curation. We used single-gene expert-reviewed databases (BRCA Exchange (BRCAEx) and the International Agency for Research on Cancer (IARC)) to carefully review such genetic variants. Only BRCAEx (https://brcaexchange.org/) summary view pathogenic variants were selected. IARC-TP53 (https://p53.iarc.fr/) somatic variants were selected only if reported from the hematopoietic system as the primary site. In addition, MET exon 14 skipping variants in NSCLC guidelines were recommended to be used as therapeutic biomarkers for Crizotinib (as category 2A). These types of genetic variants require specific laboratory validation. We collected only the skipping variants with known experimental validation [27, 28]. As a result, an expanded list for the unspecified genetic variants in the NCCN guidelines was developed (Additional file 2: Table S2).

### Data acquisition and integration

OncoKB (https://www.oncokb.org/) was used as an information source on therapeutic options (Nov, 2020). We encountered the same challenge for some genetic variants that have generic or non-specific descriptions. For example, EGFR gene exon 19 deletion and NRAS oncogenic mutations were listed as actionable variants. Only variants that mentioned the same consequence and were predicted to be deleterious by tumor-specific methods CScape (http://cscape.biocompute.org.uk/) and IntOgen (https://www.intogen.org/search) were selected (Table 1, Additional file 3: Table S3) (Nov, 2020).

**Table 1:** List of single nucleotide variants with specific consequence on OncoKB in the selected tumors

OncoKB alteration	Gene	Tumor site
Exon 14 splice mutation	MET	Non-Small Cell Lung Cancer (NSCLC)
Exon 17 mutations	KIT	Melanoma
Exon 19 deletion	EGFR	Non-Small Cell Lung Cancer (NSCLC)
Exon 19 insertion	EGFR	Non-Small Cell Lung Cancer (NSCLC)
Exon 20 insertion	EGFR	Non-Small Cell Lung Cancer (NSCLC)
Oncogenic Mutations	KRAS	Colorectal cancer, All solid tumors
Oncogenic Mutations	NRAS	Colorectal cancer, Melanoma
Oncogenic Mutations	ALK	NSCLC
Oncogenic Mutations	BRCA1	Breast Cancer (BC)
Oncogenic Mutations	BRCA2	BC
Oncogenic Mutations	ERBB2	BC, NSCLC
Oncogenic Mutations	FGFR2	All solid tumors
Oncogenic Mutations	FGFR3	All solid tumors
Oncogenic Mutations	KIT	Melanoma
Oncogenic Mutations	PIK3CA	BC
Oncogenic Mutations	TSC1	CNS
Oncogenic Mutations	TSC2	CNS
Oncogenic Mutations	ARAF	NSCLC
Oncogenic Mutations	ESR1	BC
Oncogenic Mutations	FGFR1	All solid tumors
Oncogenic Mutations	MAP2K1	Melanoma, NSCLC
Oncogenic Mutations	MTOR	All solid tumors
Oncogenic Mutations	ATM	All solid tumors
Oncogenic Mutations	CDKN2A	All solid tumors
Oncogenic Mutations	NF1	All solid tumors
Oncogenic Mutations	PTEN	All solid tumors
Oncogenic Mutations	SMARCB1	All solid tumors

Cancer genetic variant hubs were downloaded. The Oncotree model (http://oncotree.mskcc.org/) was used as a standard tissue spatial tree for all tumors. For each database, tumor primary locations were mapped to the same or nearest histopathology and cell type on the Oncotree model (Table 2). We focused on seven major cancer sites in our work: bowel, breast, brain/central nervous system (CNS), esophagus/stomach, skin, lung and pancreas. The latest COSMIC version dataset was obtained in July 2020, intOgen's latest version, and the bulk dataset of cBioPortal was downloaded in August 2020. In the cBioPortal dataset, genetic variants with no specified tumor origin sites or in samples with low tumor cellularity were deemed “problematic” and excluded.

**Table 2:** Mapped tumor sites for the used public database with the Oncotree model

Origin	Sub	NCCN	COSMIC	intOgen	cBioPortal	OncoKB
Breast	breast	breast	breast	BRCA	ACBC, BLPT, BPT, BRCA, BRCNOS, BREAST, DCIS, IDC, ILC, IMMC, MBC, MDLC, MPT, PD, SPC	Breast Cancer
Bowel	anal	anal	large_intestine > SS1 = anus	COREAD	NA	Colorectal Cancer
Bowel	rectal	rectal	large_intestine > SS1 = rectum	COREAD	READ, COADREAD	Colorectal Cancer
Bowel	colon	colon	large_intestine > SS1 = colon	COREAD	COAD, MACR, COADREAD	Colorectal Cancer
CNS or Brain	cns	cns	central_nervous_system	GBM, LGG, MB, NB, PA	AASTR, AOAST, AODG, ASTR, DIFG, GB, GBM, LGGNOS, MBL, OAST, ODG	CNS Cancer, Glioma, Embryonal Tumor
Esophagus or Stomach	esophageal	esophageal	oesophagus	ESCA	ESCC, ESCA, STES	Esophagogastric Cancer
Esophagus or Stomach	gastric	gastric	stomach	STAD	DSTAD, ESCA, ISTAD, MSTAD, STAD, STOMACH, TSTAD	Esophagogastric Cancer
Skin	melanoma	melanoma	PH = malignant_melanoma	CM	ACRM, DESM, MEL, SKCM, SKLMM	Melanoma
Pancreas	pancreas	pancreas	pancreas	PAAD	IPMN, MCN, PAAD	NA
Lung	nsclc	nsclc	lung > HS1 = non_small_cell_carcinoma	NSCLC	LUAD, LUSC, NSCLC	Non-Small Cell Lung Cancer, Lung Squamous Cell Carcinoma
Lung	sclc	sclc	lung > HS1 = small_cell_carcinoma	SCLC	NA	NA

PanCan studies were classified as well-powered while the others (single-center) were considered as small studies. A genetic variant supported by more than 5 single studies was considered as variant with multiple findings while those with fewer than 5 studies were classified as variants with a few reports (Fig. 2). Consensus was defined based on a gene’s candidacy as a tumor driver given tissue type using expert-curated resources. Census scores were developed to reflect the degree of consensus per tumor site for each gene. The score criteria is based on consistency of reviewed knowledge between a gene and a distinct cancer tissue. For this purpose, we used the Cancer Gene Census (CGC), and Candidate Cancer Gene Database (CCGD). A list of all available genes from both sources was made. Next, all tissue types were aligned with the Oncotree model (Table 2), and then multiple gene-tissue vectors were generated. CGC-based vectors were given higher ranking (consensus score = 1) and CCGD vectors were given consensus scores of 2, otherwise vectors were denoted with a value of zero. (Additional file 1: Table S1).

## Results

### Testing dataset

**Fig. 4:**
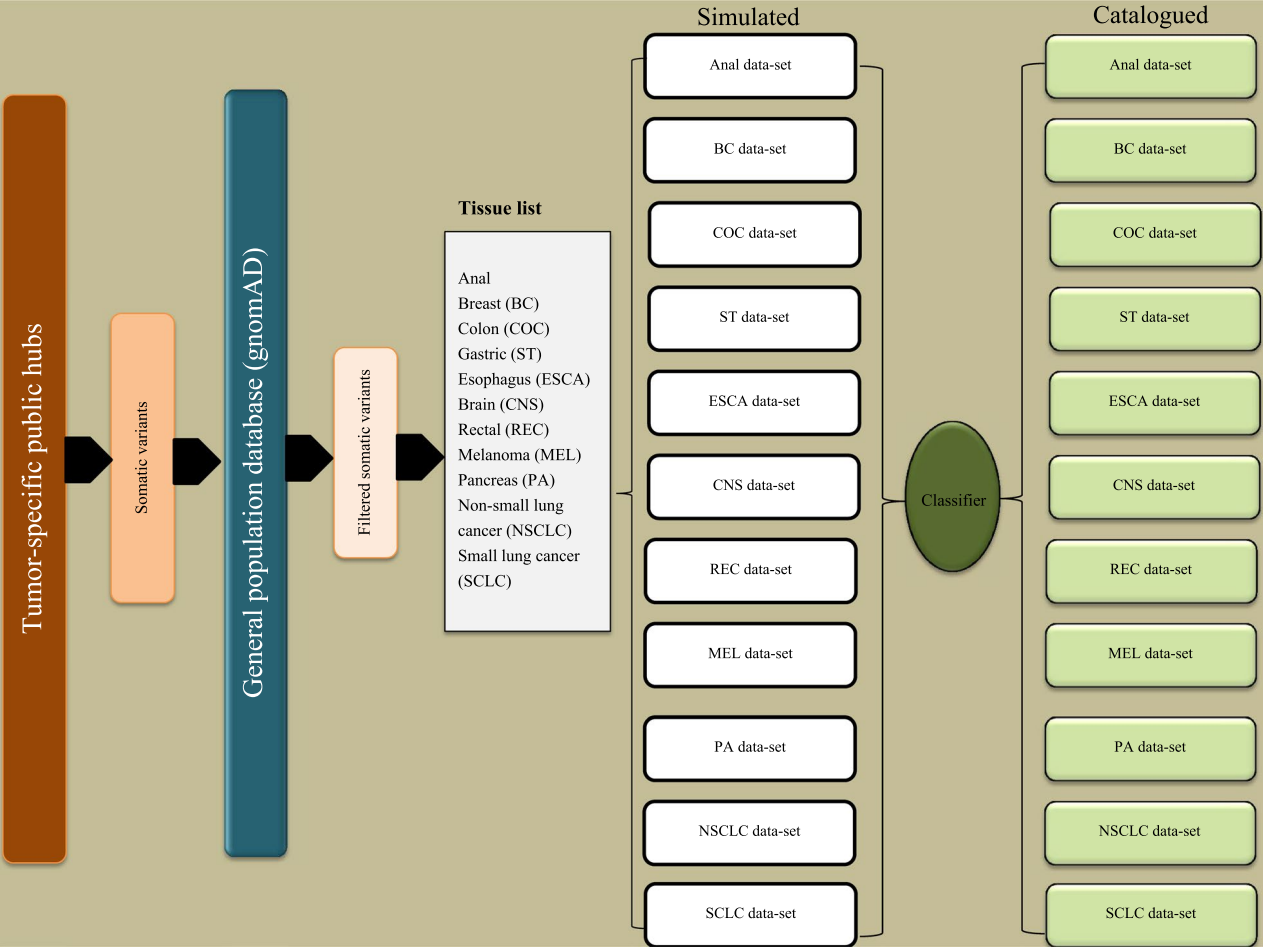
Summary of the testing data acquisition and processing

We collected all somatic variants on the aforementioned databases into a single collective list. All variants that intersected with the gnomAD (https://gnomad.broadinstitute.org/) database were removed, returning 2,952,167 somatic variants. We simulated the list with the selected tumor sites, producing eleven specific tumor-site datasets (Fig. 4).

### Classification

Every simulated dataset was interpreted and catalogued by the new algorithm. Since germline-based filtration was performed, our classifier ranked only the first three tiers of the AMP–ASCO–CAP guidelines (Table 3). The tumor tissue with the most potentially actionable genetic variants (tiers I & II) was the CNS with 413 variants while 4 tumor sites—anal, esophageal, gastric and pancreas—showed no genetic variants on tier I and had the lowest potentially actionable genetic variants (338). All of the class II variants in the tumors had level D evidence of preclinical trials or biological attestation in solid tumors. Most of the tier I variants were ranked that way because of relative discussion in the NCCN guidelines in non-specific manner and predicted as tumor drivers by intOgen and CScape (Additional file 2: Table S2).

**Table 3:** The number of ranked somatic variants per class per tumor site-specific data-set

Tumor site	I	II	III	Actionable
breast	53	338	2951776	391
colon	7	338	2951822	345
anal	0	338	2951829	338
gastric	0	338	2951829	338
esophageal	0	338	2951829	338
cns	73	340	2951754	413
sclc	19	324	2951824	343
rectal	25	330	2951812	355
melanoma	41	314	2951812	355
pancreas	0	338	2951829	338
nsclc	53	300	2951814	353
Total*	271	3636	32469930	3907

*The total is not a unique list

### Comparison

Publicly available software that build-in for the AMP–ASCO–CAP recommendations [29] is limited. Two methods were selected for comparative analysis: the Variant Interpretation for Cancer (VIC) (https://github.com/HGLab/VIC) [29] and the Cancer Genome Interpreter (CGI) (https://www.cancergenomeinterpreter.org/home) [1]. VIC, which was developed to provide clinical annotations for somatic genetic variants according to the recommended 4-tier system, was published in Aug 2019. The CGI ranks somatic genetic variants by a different 4-tier metric (Table 4). CGI provides a hierarchical structure for the tumor sites, while VIC lays out a list of tumor sites for the user input [1, 29].

**Table 4:** Somatic variant interpretation catalogs by the consensus guidelines and their equivalent on CGI

Class	VIC/AMP–ASCO–CAP	CGI
I	Strong clinical significance	Predicted driver
II	Potential clinical significance	Predicted passenger
III	Uncertain significance	Known in
IV	Benign or likely bening	Not protein-affecting or likely neutral for oncogenesis

To evaluate the new method and the other computational tools, a subset of genetic variants were collected for manually curation by clinical geneticist. Fifteen genetic variants were randomly selected from each cancer site-dataset, returning a list of 186 mutations. The curated subset resembled the ground “truth” for genetic variants clinical annotations. The selected subset had a small number of significant genetic variants (4%). Only two genetic variants were annotated as tier one, and six mutations were classified as tier two. Both tiers were recognized as true positives (TP), while the remaining variants were classified as “true” negatives (TN) for comparison purposes (Additional file 4: Table 4).

**Fig. 5:**
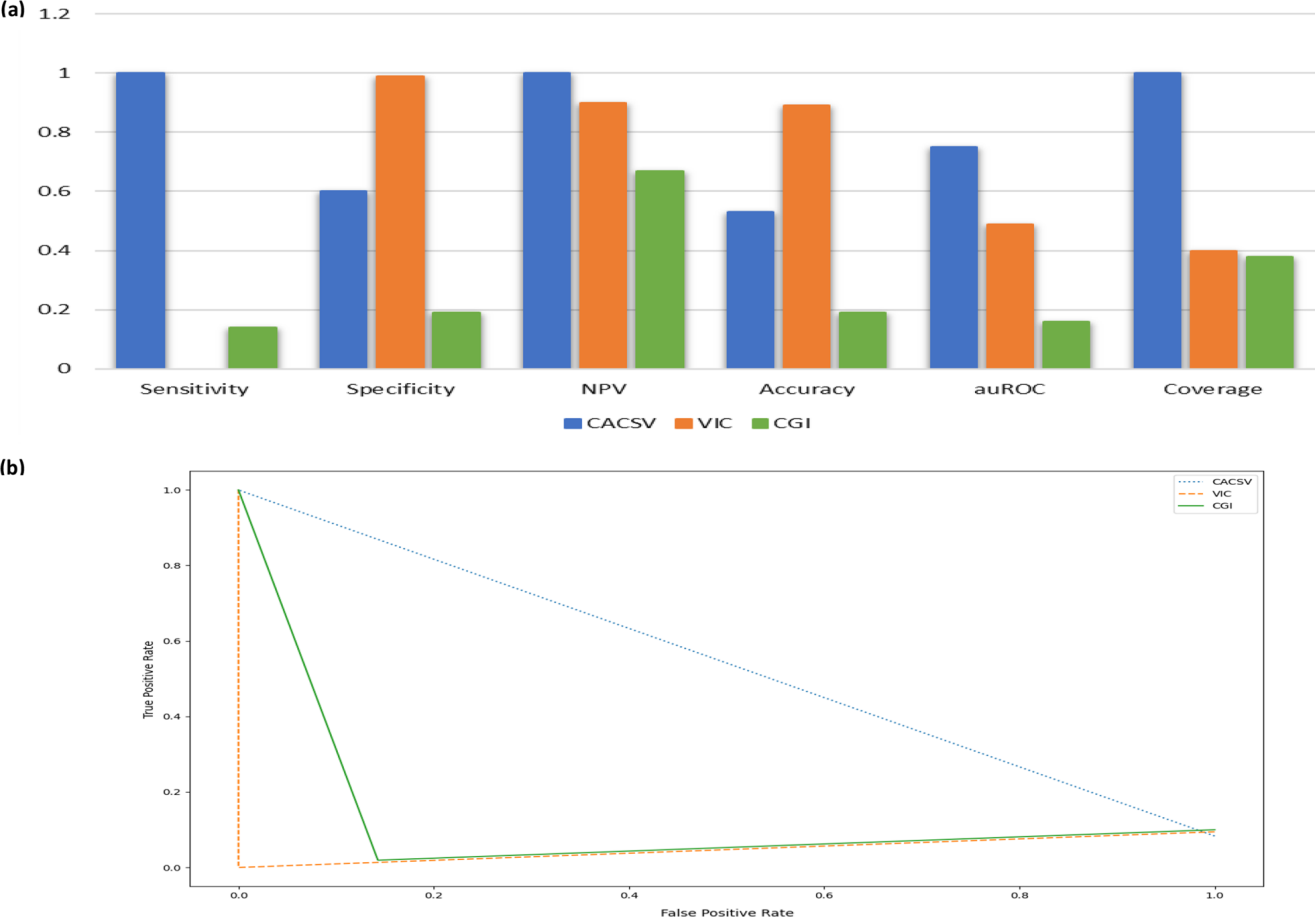
Computational tools performance evaluation. **a** Graph chart analysis for the methods’ clinical annotation performance. **b** Precision-recall trade-off curve

The results of the computational methods were inconsistent with each other. Individually, VIC and CGI did not provide clinical annotation for a significant number of the genetic variants (~ 60% of the subset). VIC classification had the highest accuracy and specificity (0.89, 0.99). Our method showed the best sensitivity, availability of clinical annotation (coverage), area under the receiver operating characteristic (auROC), and negative predictive value (NPV). The positive predictive value (PPV) was not considered due to the limited number of TP (8 variants). The curated subset had an imbalanced ratio of TP and TN (1:23) which could be challenging for methods evaluation. To examine the effect of this disproportional ratio on the ability an algorithm to maintain a good precision-recall trade-off, the area under the precision-recall curve (auPRC) was used (Table 5, Fig. 5).

**Table 5:** Computational methods evaluation in comparison to the clinically-classified subset

Method	Sensitivity	Specificity	NPV	Accuracy	auROC	Coverage
CACSV	1	0.6	1	0.53	0.75	1
VIC	0	0.99	0.90	0.89	0.49	0.4
CGI	0.14	0.19	0.67	0.19	0.16	0.38

Negative predictive value (NPV) is the proportion of true negative to all negatives (true or false), and coverage appraise the availability of clinical annotation by the method

## Discussion

The application of parallel sequencing in oncology for research and diagnostics has resulted in the creation of immense size of databases [30–32]. The availability of multiple, expert-reviewed sources for onco-genetics has deepened our understanding of tumorigenesis and expanded the tumor-molecular networks. In addition, the development of genetic variants predictive models and classifiers has helped in filtering, classifying, and interpreting cancer genetic variants in clinical settings. Nonetheless, observable inconsistencies between analytical workflows have created a demand for a clinical standard [21]. The AMP–ASCO–CAP recommendations provide the first standardized approach for cataloguing cancer genetic variants. The guidelines suggest extrapolating the ACMG/AMP recommendations [33] for interpreting constitutional variations and proposing a new algorithm for tumor-specific variants classification. In this study, the AMP–ASCO–CAP recommendations were incorporated and applied to publicly available somatic variants.

In our attempt to build-in the recommended guidelines, we needed to define some of the requirements for achieving amenable adherence and for reducing ambiguity. The AMP–ASCO–CAP recommendations suggested the use of medical guidelines to provide a source of accurate clinical utility; however, multiple oncological professional guidelines are used in clinical practice. The list includes: American Society of Clinical Oncology (ASCO), European Society for Medical Oncology (ESMO), and National Comprehensive Cancer Network Clinical Practice Guidelines in Oncology (NCCN Guidelines®). Understandably, subtle yet detectable variations in regional and institutional guidelines exist, in particular with regards to the guidelines descriptions of genetic screening and treatment options [34]. For example, eight guidelines suggested treatments for breast cancer (BC) patients with BRCA variants or with high familial risk for developing BC. Three guidelines suggested the use of platinum therapy as neoadjuvant therapy for BRCA-mutated metastatic BC for women under the age of 40 while the NCCN guidelines recommended the use of the PARP inhibitor olaparib for treatment of BRCA-mutated HER2-negative BC [34]. Ideally, congruent clinical protocols would provide improved and standardized healthcare delivery. In this study, we chose only a single oncological professional guideline.

The ground “truth” subset had a considerably small size relative to the “original” cancer datasets and only 8 genetic variants with known/potential clinical significance or TP. Additionally, a significant number of genetic variants (~ 60%) had no clinical interpretation by the used in silico algorithms, except our method. However, the lack of clinical annotations in bioinformatics tools or cancer genomic databases is fairly common in cancer genetics. VIC, CGI, and our method performances were significantly disparate on the curated genetic variants. VIC had the highest accuracy (0.89), however it only provided classification for 73 genetic variants (of 186). Our method provided full clinical annotation for the subset and had the best sensitivity and auROC. CGI was not designed to follow the 2017 guidelines (Table 4), however, its inclusion in the results may reflect the potential discrepancies when different classification systems are implemented.

The discrepancy between our classifier and other methods is not unexpected. The CGI ranks somatic genetic variants based on level of evidence from manually curated genomic databases, including ClinVar [31]. In addition, tumor-driver genes were predicted using the analysis of large cancer cohorts such as The Cancer Genome Atlas and the International Cancer Genome Consortium (https://icgc.org/) [35] and then verified by the availability of experimental or computational validation. While ClinVar provides clinical and experimental evidence for germline genetic variants, it lacks information concerning somatic alterations. The AMP–ASCO–CAP guidelines suggested the use of ClinVar for tumor germline variants in the current state [21]. The guidelines also do not count in silico analysis for any cohort size or the score of predictive models as sufficient evidence for clinical action [21]. VIC uses prediction scores from multiple methods and uses consistency threshold of at least four in their algorithm criteria. We used only somatic genetic variant predictive models (as they are shown to have better false positive rates [20]) and we used them only to verify oncogenic variants in genes that are reported in the professional guidelines. We suggest caution in interpreting these variants as they may require additional experimental verification. In addition, VIC provides a more dynamic option for variant interpretation through manual inputs by users which would change variant classifications from the “default” settings.

We acknowledge some limitations in the CACSV database. First, there was no consideration for tumor pathway involvement. Cancer molecular networks are complex and frequently evolving; a good implementation of tumor-specific networks would allow for better variant classification. In addition, consensus guidelines recommend fine mapping to the nearest cell type: the analytical principal in the interpretation of variants with unknown significance. We plan to address these shortcomings in future updates of the CACSV. Also, oncologists’ professional guidelines provide a range of therapeutic biomarkers including gene expressions, fusions and translocations while our approach covers only small DNA genetic variants as some of the other biomarkers are not easily detectable by current parallel sequencing methods and are screened by other molecular assays. Prospective CACSV releases will cover other complex biomarkers: the availability of tumor-specific, FDA-approved treatments or investigative therapies is a key criterion in the AMP–ASCO–CAP guidelines. In our current work, OncoKB was used for existing information about active or approved treatments. Adapting consensus guidelines should provide a global knowledgebase of all available treatments. We will include other international resources in the future, e.g. the University Hospital Medical Information Network (UMIN) (https://www.umin.ac.jp/english/) [36]. We are also working on developing a user graphical interface to the CACSV to reach a wider range of users that would also provide a sharable genetic variants hub for the clinical and research communities.

## Conclusion

We’ve developed a simulated database (CACSV) for multiple tumors that provides clinical annotations for publicly available cancer genetic variants by a new algorithm that incorporates AMP–ASCO–CAP recommendations. The fully classified dataset is available as built-in formats (JSON) by most bioinformatics pipelines in clinical and research settings. CACSV is freely available at https://github.com/tsobahytm/CACSV/tree/main/dataset.

## Supplementary Information

**Additional file 1: Table S1**. List of all cancer consensus genes against their tissue type involvement.

**Additional file 2: Table S2.** List of unspecified genetic variants in the NCCN guidelines that were experimentally validated, curated by gene-specific database, or predicted as deleterious by two somatic variant predictors (intOgen & CScape).

**Additional file 3: Table S3**. List of OncoKB unspecified variants that have the same described mutational consequence and are predicted to be deleterious by two somatic variant predictors (intOgen & CScape).

**Additional file 4: Table S4**. Random subset of the simulated data classified manually and by the computational methods.

## Data Availability

The final datasets are available at GitHub (https://github.com/tsobahytm/CACSV/tree/main/dataset). All Python programs used to generate the final output are also available (https://github.com/tsobahytm/CACSV/tree/main/scripts). The README file contains more details about each program and dataset. All somatic variant databases or tools used in the analysis and comparative testing can be found via the following URLs. COSMIC: https://cancer.sanger.ac.uk/cosmic. cBioPortal: https://www.cbioportal.org/. CGC: https://cancer.sanger.ac.uk/census. CCGD: http://ccgd-starrlab.oit.umn.edu/. BRCAEx: https://brcaexchange.org/. IARC-TP53: https://tp53.isb-cgc.org/. OncoKB: https://www.oncokb.org/. CScape: http://cscape.biocompute.org.uk/. IntOgen: https://www.intogen.org/search. gnomAD: https://gnomad.broadinstitute.org/. VIC: https://github.com/HGLab/VIC. CGI: https://www.cancergenomeinterpreter.org/home.

## Abbreviations

AMP: The Association for Molecular Pathology
ASCO: American Society of Clinical Oncology
CAP: College of American Pathologists
CACSV: Clinically actionable cancer somatic variants
NGS: Next-generation sequencing
CNVs: Copy number variations
TCGA: The cancer genome atlas
COSMIC: Catalogue of somatic mutations in cancer
PCT: Personalized cancer therapy
NCCN: National Comprehensive Cancer Network Clinical Practice Guidelines in Oncology
OncoKB: Precision oncology knowledge base
CGC: Cancer gene census
CCGD: Candidate cancer gene database
SNVs: Single genetic variants
AML: Acute myeloid leukemia
IARC: International Agency for Research on Cancer
BRCAEx: BRCA exchange
CNS: Central nervous system
VIC: Variant interpretation for cancer
CGI: Cancer genome interpreter
ACMG: American College of Medical Genetics
UMIN: University Hospital Medical Information Network
